# Identification of a 3-aminoimidazo[1,2-a]pyridine inhibitor of HIV-1 reverse transcriptase

**DOI:** 10.1186/1743-422X-9-305

**Published:** 2012-12-11

**Authors:** Daniel Elleder, Thomas J Baiga, Rebecca L Russell, John A Naughton, Stephen H Hughes, Joseph P Noel, John AT Young

**Affiliations:** 1 The Salk Institute for Biological Studies, Nomis Center for Immunobiology and Microbial Pathogenesis, 10010 North Torrey Pines Road, La Jolla, CA 92037, USA; 2 The Salk Institute for Biological Studies, Howard Hughes Medical Institute, Jack H. Skirball Center for Chemical Biology and Proteomics, 10010 North Torrey Pines Road, La Jolla, CA, 92037, USA; 3 HIV Drug Resistance Program, National Cancer Institute-Frederick, Frederick, MD, 21702, USA; 4 Current Address: Institute of Molecular Genetics, Academy of Sciences of the Czech Republic, Videnska 1083, Prague, Czech Republic; 5 EMD Millipore Corporation, Bioscience Business Unit, 10394 Pacific Center Court, San Diego, CA, 92121, USA

**Keywords:** HIV-1, NNRTI, Inhibitor

## Abstract

**Background:**

Despite the effectiveness of highly active antiretroviral therapy (HAART), there remains an urgent need to develop new human immunodeficiency virus type 1 (HIV-1) inhibitors with better pharmacokinetic properties that are well tolerated, and that block common drug resistant virus strains.

**Methods:**

Here we screened an in-house small molecule library for novel inhibitors of HIV-1 replication.

**Results:**

An active compound containing a 3-aminoimidazo[1,2-a]pyridine scaffold was identified and quantitatively characterized as a non-nucleoside reverse transcriptase inhibitor (NNRTI).

**Conclusions:**

The potency of this compound coupled with its inexpensive chemical synthesis and tractability for downstream SAR analysis make this inhibitor a suitable lead candidate for further development as an antiviral drug.

## Background

Despite more than 25 years of research, drug treatment of HIV infection remains a major therapeutic challenge. The most effective regimen for treating HIV-1 infection is highly active antiretroviral therapy (HAART) that usually consists of a non-nucleoside reverse transcriptase inhibitor (NNRTI) or protease inhibitor together with two nucleoside (or nucleotide) reverse transcriptase inhibitors (NRTIs).

Over 50 NNRTIs have been described to date [1]. Five of these NNRTIs have been approved by the United States FDA for the clinical treatment of HIV infection and AIDS. The first generation NNRTIs used are efavirenz, nevirapine and delavirdine. However, the rapid emergence of virus drug-resistant virus limited the effectiveness of these drugs. The second-generation NNRTIs, etravirine and rilpivirine, are more active against both drug-sensitive and resistant virus strains [1]. However, despite this progress there is a continued need for the development of novel NNRTIs which have better pharmacokinetic properties and inhibit common drug-resistant virus strains [1].

Here we screened a chemical library to identify novel inhibitors of HIV-1 replication. We report the identification of a lead compound containing a 3-aminoimidazo[1,2-a]pyridine scaffold that acts as an NNRTI, with inherent features that render it attractive for further development as an antiviral drug.

## Results

The Salk Institute in-house small molecule collection is a chemically diverse set of synthetic compounds based on divergent design principles. Fundamental to this compound collection is a Diversity-Oriented Synthesis (DOS) approach [2] that is pharmacophore or scaffold-centric, with emphasis on the use of multi-component synthetic reactions (MCRs) [3] and a variety of post-MCR transformations or reaction cascades [4]. The diversity criteria are biased by Lipinski’s ‘Rule of 5’ [5], ADME-Tox filtering, exclusion of reactive substituents [6] and incorporation of masked functional groups within scaffold side chains to enable additional chemical manipulations via forward or reverse chemical genetic screening methodologies [7]. The synthetic economy realized by such a library design permits the inclusion of rare and exotic building blocks, which further enhances the chemical diversity of the library and mitigates the limitations of library size, scope, and scale. An additional attribute of the library design, due in large part to the economics of syntheses, is the ability to rapidly determine structure-to-activity relationships (SAR) early in the evaluation of lead compounds. Each pharmacophore-based library subset can be readily expanded to further explore the relevant target space, enabling both lead optimization and lead evolution (or scaffold hopping) to proceed in parallel.

Four hundred and eighty compounds, constituting a cross-section of the Salk library, were screened in a plate-based assay to identify small molecules that inhibit a single cycle of replication by a VSVg-pseudotyped HIV-1 vector (pNL4-3LucR+E-) encoding firefly luciferase [8]. This vector is competent for only the early steps of retroviral replication leading up to viral DNA integration and gene expression. Human 293T cells were pretreated for 1 hr with 1 μM final concentration of the individual compounds and then challenged with the VSVg pseudotyped HIV-1 vector in the continued presence of that compound. After 24 hrs, the luciferase activity of each sample was determined.

**Figure 1:**
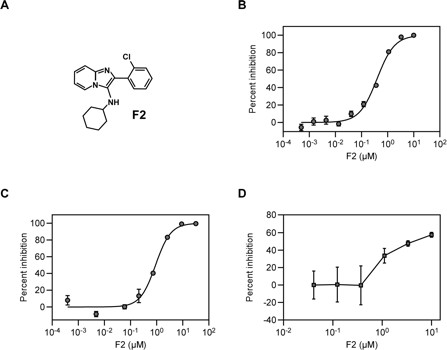
**The F2 compound blocks an early step of HIV-1 replication. A**. Structure of compound **F2**. **B**.Dose–response curve of **F2** in 293T cells challenged with the VSVg pseudotyped pNL4-3lucR+E- vector. The effect of the compound on infection was determined by measurement of virus-encoded firefly luciferase activity. The experiment shown, performed with triplicate samples, is representative of six independent experiments. **C**. Dose–response curve of **F2** in CEM-GFP cells challenged with the pLai3Luc2 HIV-1 vector [10].The numbers of GFP positive cells at 2 days post-infection were determined by flow cytometry and the experiment shown, performed with duplicate samples, is representative of two independent experiments. **D**. Dose–response curve of **F2** in human PBMCs challenged with the NL4-3 Nef+ IRES rluc vector encoding renilla luciferase activity, measured at 5 days post-infection. The experiment shown was performed with eight replicate samples. The error bars (panels B-D) represent the standard errors of the mean.

A single compound, **F2** (mol. wt. 325.83 g/mol), was identified that decreased luciferase activity by more than 50% under these conditions. **F2** originated from a sub-library representing the 3-aminoimidazo[1,2-a]pyridine scaffold (Figure 1A) derived from the 3-component Groebke condensation reaction [9]. **F2** was re-synthesized and purified at a larger scale for the subsequent experiments described in this report. Luciferase-based infectivity assays in the presence of increasing concentrations of **F2** yielded dose response curve with a mean EC_50_ ± standard deviation of 0.387 ± 0.046 μM (*n* = 6) (Figure 1B). Cytotoxicity for **F2** was determined 24 hrs after the compound was added to mock infected cells; the CC_50_ value was 34.1 ± 2.4 μM (*n* = 3), selectivity index = 88.1.

Because the initial screen was performed by scoring firefly luciferase reporter gene expression from a VSVg pseudotyped virus vector, it was possible that the **F2** compound inhibited either an early step of HIV-1 replication, VSVg-mediated cellular entry, or firefly luciferase reporter activity. To exclude possible effects on VSVg-specific cellular entry and firefly luciferase activity, **F2** was tested for its ability to block infection of CEM-GFP lymphocytic indicator cells by a replication-competent HIV-1 vector with a wild-type CXCR4-tropic HIV-1 envelope glycoprotein [10]. In this assay, infection by the wild-type virus leads to the expression of a GFP reporter gene in a Tat-deficient HIV-1 provirus that is resident in the CEM-GFP cell line. The measured EC_50_ in these experiments (0.862 ± 0.088 μM; *n* = 2) (Figure 1C), was similar to the value obtained with the VSVg-pseudotyped virus. The CC_50_ value obtained with the CEM-GFP cells was 25.4 ± 2.1 μM (*n* = 2), selectivity index = 29.5. Similarly, **F2** inhibited infection of primary human peripheral blood mononuclear cells (PBMCs) by a replication-competent HIV-1 vector (NL4-3 Nef + IRES rluc) with a measured EC_50_ of 0.865 ± 0.222 μM (Figure 1D) in the absence of cell toxicity, up to 10μM of compound tested (data not shown, selectivity index > 11.6). Taken together, these results suggested that **F2** blocks an early step of HIV-1 replication.

**Figure 2:**
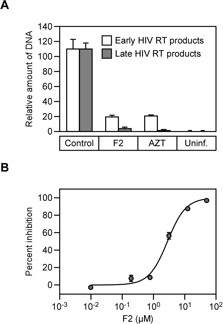
**The F2 compound inhibits HIV-1 reverse transcriptase. A**. Effects of **F2** (5 μM) treatment on the synthesis of early and late viral DNA in human 293T cells challenged with the VSVg-pseudotyped HIV-1 vector, measured at 24 hours post-infection. AZT (5 μM) was used as a reference compound. The values represent amounts of DNA relative to control, untreated cell populations, with error bars showing standard deviations from three independent real-time quantitative PCR assays. **B**. Effect of compound F2 on HIV-1 RT activity *in vitro*, determined by measuring the [alpha32P]-dTTP incorporation. The experiment shown, performed with duplicate samples, is representative of two independent experiments. with error bars representing standard errors of the mean.

A quantitative real-time PCR-amplification approach was used to determine whether **F2** treatment blocks viral DNA synthesis. Total DNA was isolated from cells 24 hrs post infection and quantified using primers and probes specific for early and late HIV-1 reverse transcription products [11]. **F2** (5 μM) added 1 hr before infection blocked the synthesis of both early and late viral DNA products (Figure 2A), suggesting that this compound might inhibit HIV-1 reverse transcriptase. To directly test that possibility, an *in vitro* assay was used to directly test the effect of **F2** on recombinant purified HIV-1 reverse transcriptase (RT) activity. **F2** potently inhibited HIV-1 RT activity *in vitro* in a dose-dependent manner with an IC_50_ = 2.554 ± 0.365 μM (*n* = 2) (Figure 2B). Although the 50% inhibitory concentration of the **F2** compound was higher in the *in vitro* experiment with purified HIV-1 RT than in the cellular infectivity assays, this type of result is seen frequently with NNRTI inhibitors [12].

**Figure 3:**
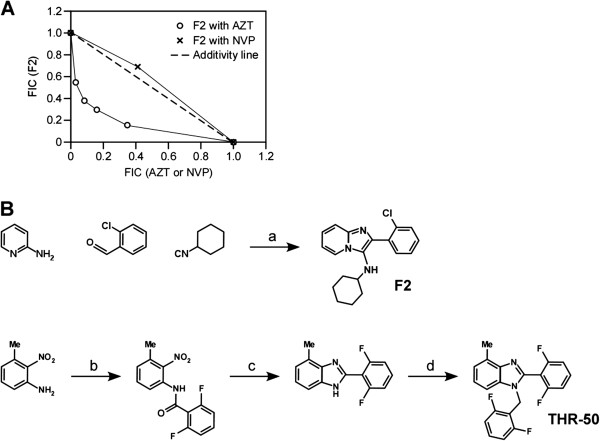
**The F2 compound is a NNRTI. A**. Isobologram analysis of the effect of combining **F2** with either AZT or NVP in human 293T cells challenged with the VSVg-pseudotyped HIV-1 vector. Depicted are isobologram plots for the 90% inhibitory level (EC_90_). The dashed line indicates the values expected for an additive effect. Values below and to the left of the line indicate synergistic effects. FIC, fractional inhibitory concentration. **B**. Structure and synthesis of compound **F2** compared to THR-50 [15]. Reagents: (a) 0.1 M in 1,2-dichloroethane – 2,2,2-trifluoroethanol (1:1), 5.0 mol% Sc(OTf)_3_, rt for 96h or microwaved at 140°C for 5min, >90% yield; (b) 2,6-F_2_BzCl, THF/pyridine (5:1), rt for 2h, 55% yield; (c) Fe, AcOH, reflux, 97% yield; (d) 2,6-F_2_BnBr, NaH, THF, rt overnight, 75% yield.

The two main classes of existing HIV-1 RT inhibitors (the NRTIs and NNRTIs) act synergistically, especially when used at high inhibitory concentrations [13,14]. To determine whether there is a similar synergy seen with **F2**, this compound was tested along with the NRTI 3’-azido-3’-deoxythymidine (AZT) and with the NNRTI nevirapine (NVP) in combinations at several fixed molar ratios and over a range of serial dilutions [13]. The resulting isobologram plots demonstrated that **F2** exhibits synergy with AZT and additivity with NVP in a cellular infectivity assays, suggesting that **F2** blocks HIV-1 reverse transcriptase activity by acting as a NNRTI (Figure 3A).

Since it is desirable for a candidate antiretroviral to be effective against HIV-1 variants resistant to current NNRTIs, the resistance profile of **F2** was tested against four commonly encountered NVP-resistant HIV-1 mutant variants (K103N, V106A, Y188L, Y181C) [1]. The mutant vectors used were based on the HIV-1 NL4-3 strain pseudotyped with VSVg and infection of 293T cells was monitored using a standard single cycle luciferase-based cellular infectivity assay. **F2** inhibited infection by the mutant viral vectors, albeit at reduced levels compared to the wild-type virus (EC_50_ values were reduced between 8- to more than 120-fold; Table 1). The resistance profile of **F2** was similar to NVP (Table 1), reinforcing the idea that this compound acts as a NNRTI.

**Table 1:** Resistance profile against wild type and RT mutant VSV-G pseudotyped HIV-1 vectors

**Inhibitor**	**Antiviral EC**_**50**_**(μM)**^**a, b**^					**CC**_**50**_**(μM)**^**c**^
	wt	Y188L	Y181C	V106A	K103N	
F2	0.165 ± 0.039	> 20	1.676 ± 0.271	1.275 ± 0.357	3.233 ± 0.127	34.1 ± 2.4
		[>121.1]	[10.2]	[7.7]	[19.6]	
NVP	0.019 ± 0.002	> 20	2.879 ± 0.006	1.260 ± 0.159	0.841 ± 0.049	> 50
		[>1052.6]	[151.5]	[66.3]	[44.2]	
AZT	0.014 ± 0.003	0.011 ± 0.005	0.006 ± 0.0	0.015 ± 0.001	0.014 ± 0.002	> 50
		[0.8]	[0.4]	[1.1]	[1.0]	

^a^ Values are averages ± standard deviations from two independent experiments performed in duplicate in 293T cells.

^b^ The viruses were produced by cotransfection of the pNLNgoMIV R+E-.luc wild type or RT mutant plasmids and VSVg expressing plasmid. The values in brackets indicate the fold change in EC_50_ for mutant relative to wild type.

## Discussion

In this report we describe a new lead candidate NNRTI inhibitor that has activity against wild-type and some of the common drug-resistant variants of HIV-1 reverse transcriptase. Serendipitously, this new lead scaffold is structurally isosteric with THR-50, a benzimidazole-based NNRTI [15] (Figure 3B). The imidazo[1,2-a]pyridine pharmacophore of **F2** can be realized by three instances of bioistosteric replacement on the benzimidazole scaffold; (1) N1 nitrogen is converted to carbon, (2) bridgehead carbon adjacent to C7 is converted to nitrogen, and (3) benzylic methylene (CH_2_) is converted to NH. These subtle changes in the inhibitor core [15] slightly enhance the physical properties, create an additional H-bond donor interaction, and greatly simplify the synthesis of analogs for optimization studies.

While the THR-50 compound is the more potent inhibitor against wild-type and drug-resistant variants of HIV-1 reverse transcriptase, the **F2** compound has several distinct advantages for downstream drug development. Specifically, **F2** features an imidazo[1,2-a]pyridine core scaffold that is generated in a simple, inexpensive, single chemical transformation that belongs to a special class of diversity generating chemistries known as Multi-Component Reactions (MCR), leading to high product yield and purity. By contrast, the benzimidazole core scaffold of the NNRTI THR-50 results from a three-step chemical process (Figure 3B) resulting in an overall yield of 40% and requiring purification operations after each reaction step. Furthermore, the number of possible analogs to **F2**, based on commercially available building blocks, is on the order of 10^6^ compounds, versus 10^3^ for THR-50. These two molecular structures are further distinguished by the addition of an H-donating group (NH) for additional binding interactions as well as lowering clog P and improving the pharmacological properties. Therefore, the lead **F2** compound is an excellent candidate for further development as an antiviral drug through small molecule SAR and structure-guided design.

## Methods

### Chemistry

The Salk Small Molecule Screening Collection was designed and synthesized in-house using known methods. The imidazo[1,2-a]pyridines were prepared in a 96 well microplate (Eppendorf, 2.2 ml deepwell, polypropylene) as a combinatorial matrix where 2-amino pyridine were dispensed to all wells, aromatic aldehydes were dispensed as row reagents, isonitriles were dispensed as column reagents, and lewis acid catalyst scadium (III) triflate was dispensed to all wells. The amine and isonitriles were prepared as 0.5 M stock solutions in 1,2 dichloroethane (DCE), and the aldehydes and lewis acid were prepared as 0.5 M and 0.025 M (respectively) stock solutions in 2,2,2-trifluorothanol (TFE). For a given well: 200 μl of 0.5M 2-amino pyridine in DCE, 200 μl of 0.5 M aromatic aldehyde in TFE and 200 μl of 0.025 M scandium (III) triflate in TFE were added and mixed on a plate shaker for 15 minutes at room temperature. To this solution was added 200 μl of 0.5 M isonitrile in TFE and 200 μl of (1:1 v/v) DCE-TFE solvent. Final reaction volume was 1.0 ml and theoretical concentration was 0.1 M. The plate was sealed (heat seal, foil) and allowed to shake for 5 days at room temperature. After 5 days, the crude reaction mixture was evaporated to dryness and resuspended in 1.0 ml dichloromethane (DCM). This crude reaction mixture was transferred to a filter plate charged with a cocktail of scavenging resins (PS-Trisamine, PS-NCO, PS-TsNHNH_2_) and silica gel and allowed to gravity filter after 2 hr. Each well was rinsed with 2.0ml DCM, the fractions combined and were concentrated to dryness. Resultant purity-enriched compound was resuspended in 1.0 ml DMSO (100%) for a theoretical stock solution of 100 mM. From the 100 mM master (or mother) plate, 10 mM compound in DMSO daughter plates were generated and distributed for assays. Analytical quality control was performed via high-throughput LCMS on an Agilent 1100 HPLC-LC/MSD Trap XCT MS employing ballistic gradients on Synergi Fusion RP C18 (Phenomenex) columns and acetronitrile-water solvent system. Hits were resynthesized on a 1.0 mmol scale, purified by preparative liquid chromatography and characterized by NMR and LCMS.

### DNA constructs and virus production

The VSVg-pseudotyped HIV-1 vector was generated by transient transfection of human 293T cells (American Type Culture Collection No. CRL-11268), with plasmid pNL4-3LucR+E- [8] and pMD.G plasmid that expresses the VSVg glycoprotein. The titer was determined by antibody staining for Gag (p24) expressing cells following infection. The LAI-based replication-competent HIV-1 vector was generated by transient transfection of human 293T cells with plasmid pLai3Luc2 [10]. The NL4-3 Nef+ IRES rluc vector encoding renilla luciferase was derived from NL4-3 Nef+ IRES eGFP vector [16] by replacement of the eGFP open reading frame with renilla luciferase (kindly provided by the Chanda laboratory, Sanford-Burnham Medical Institute, La Jolla). The set of vectors harboring the RT mutations and corresponding wild type form (pNLNgoMIV R+E-.luc) were based on the HIV-1 NL4-3 strain; the RT region of these vectors was derived from the BH10 isolate [17]. The vectors were pseudotyped with VSVg by cotransfection with pMD.G plasmid in 293T cells. To generate the cells that express luciferase from established HIV proviral DNA, 293T cells were infected with the VSVG pseudotyped HIV-1 pNL4-3LucR+E- vector and passaged for two weeks to remove any remaining unintegrated viral DNA.

### Tissue culture-based infectivity assays

Ten thousand human 293T cells were plated in 80 μl medium in each well of 96-well tissue culture plate. Next day, 10 μl of each diluted compound was added to reach the desired concentration and incubated at 37°C for 1 hour. A 10 μl aliquot of medium containing the VSVG pseudotyped HIV-1 vector (multiplicity of infection of 0.1-0.5) was then added to each well. Twenty-four hours after viral challenge, the medium was carefully removed and 60 μl of the Bright-Glo reagent (Promega, Madison, WI) diluted 1:1 in PBS was added to lyse the cells and provide the luciferin substrate for virus-encoded firefly luciferase. After several minutes the luminescence associated with each sample was measured, and served as readout to quantify virus infectivity in each well. The best fitted curves and EC_50_ values were calculated using Prism 4 software (GraphPad Software, San Diego). The degree of synergism between screen compounds and AZT (Sigma, St. Louis, MO) were determined by testing the compounds in the infectivity assay individually and in combinations at a fixed molar ratio over a range of serial dilutions [13]. The data were then analyzed by the isobologram technique, which evaluates the compound interactions by a dose-oriented geometric method [13,14]. Cytotoxicity of the compounds was measured 24 hours after treatment of mock-infected cells by adding an equal volume of CellTiter-Glo (Promega, Madison, WI) and reading luminescence.

In the assays employing the replication-competent pLai3Luc2 HIV-1 vector, CEM-GFP lymphocytic indicator cells [18] were pretreated with **F2** for 1 hr in 24-well plates, challenged with the virus by spinoculation at 1,200 × g for 1 hr and the number of GFP positive cells was determined by flow cytometry two days later using FACScan (Becton-Dickinson, Franklin Lakes, NJ).

Human PBMCs from an uninfected individual were obtained from the UCSD Center for AIDS Research. Samples were collected with written informed consent under Salk Institutional Review Board Protocol # 10–004. PBMCs were activated in PBMC Growth Medium (RPMI 1640 with 15% FBS, Pen/Strep, 25 mM HEPES, 100 U/ml IL-2) supplemented with 5 μg/ml PHA-P. Two days after activation, the PBMC’s were maintained in the same medium (without PHA-P) for 3 more days. Aliquots of 2x10^4^ PBMCs were then plated in 80 μl PBMC Growth Medium in each well of white 96 well plates (CoStar) and returned to the incubator overnight. Serial 3-fold dilutions of compound F2, ranging from 100 μM to 1.37 μM in concentration, were made up in 10% DMSO. Eight replicate samples were then set up, with 10 μl aliquots of the serially-diluted compounds added to the cells, and preincubated at 37°C for one hour prior to virus challenge. The cells were then challenged with 10 μl per well of the NL4-3 Nef+ IRES rluc virus vector bringing the total volume in each well to 100 μl final. The plates were incubated at 37°C for five days and then viral infection was monitored by adding 100 μl per well of Renilla-Glo Luciferase Reagent (Promega) and measuring the resultant luciferase activities using a Topcount-HTS (PerkinElmer). Cell toxicity was assessed on a duplicate plate of samples by adding 100 μl Cell-Titer Glo Reagent (Promega) and reading on a Topcount-HTS.

### Real-time PCR quantitation of viral DNA

Total cellular DNA was harvested 24 hours post infection with the AccuPrep genomic DNA extraction kit (Bioneer Life Science Corp., Rockville, MD). The amount of viral DNA products was quantified by real-time PCR on ABI Prism 7900 Sequence Detection System (Applied Biosystems, Foster City, CA) with the following primers and probes: early HIV-1 reverse transcripts with primers ert2f, ert2r, probe ERT2, late HIV-1 reverse transcripts with primers MH531, MH532, probe LRT-P [11]. To normalize for the number of cellular DNA equivalents in the samples, a single-copy locus in the PBGD gene was amplified with primers PBGD1 (5’-AAGGGATTCACTCAGGCTCTTTC), PBGD2 (5’-GGCATGTTCAAGCTCCTTGG) and probe PBGD-P (5’-VIC-CCGGCAGATTGGAGAGAAAAGCCTGT-MGBNFQ).

### HIV-1 RT *in vitro* enzyme assay

The assay was adapted from references [14,19]. HIV-1 RT (0.5 units; Ambion, Austin, TX) was incubated with different concentrations of the **F2** compound for 5 minutes at room temperature. A template-primer mixture was then added to a final concentration of 5 μg/ml oligo(dT)_20_, 10 μg/ml poly(rA), 1.25 μM [α-32P]dTTP and 10 μM dTTP. The sample was incubated at 37°C for 60 minutes. Aliquots of the reaction were spotted on DEAE paper, washed twice with 2xSSC buffer (300 mM NaCl, 30 mM sodium citrate) and once with 95% ethanol, dried and exposed to a PhosporImager screen (Molecular Dynamics, Sunnyvale, CA). The screens were scanned by a FLA-5100 instrument (Fujifilm Life Science, Stamford, CT) and the amount of incorporated labeled phosphate was used to quantify the RT activity. The best fitted curves and IC_50_ values were calculated using Prism 4 software (GraphPad Software, San Diego).

## Competing interests

The authors declare that they have no competing interests.

## Authors’ contributions

DE, TB, RR, and JAN performed the experiments. DE, TB, SH, JPN, and JATY conceived the study and designed the experiments. DE, TB, SH, and JATY analyzed the data and drafted the manuscript. All authors read and approved the final manuscript.
